# In vivo synaptic activity-independent co-uptakes of amyloid β_1–42_ and Zn^2+^ into dentate granule cells in the normal brain

**DOI:** 10.1038/s41598-019-43012-0

**Published:** 2019-04-24

**Authors:** Haruna Tamano, Naoya Oneta, Aoi Shioya, Paul A. Adlard, Ashley I. Bush, Atsushi Takeda

**Affiliations:** 1 Department of Neurophysiology, School of Pharmaceutical Sciences, University of Shizuoka, 52-1 Yada, Suruga-ku, Shizuoka, 422-8526 Japan; 2 The Florey Institute of Neuroscience and Mental Health, The University of Melbourne, Parkville, VIC 3052 Australia

**Keywords:** Molecular neuroscience, Alzheimer's disease

## Abstract

Neuronal amyloid β_1–42_ (Aβ_1–42_) accumulation is considered an upstream event in Alzheimer’s disease pathogenesis. Here we report the mechanism on synaptic activity-independent Aβ_1–42_ uptake *in vivo*. When Aβ_1–42_ uptake was compared in hippocampal slices after incubating with Aβ_1–42_, *In vitro* Aβ_1–42_ uptake was preferentially high in the dentate granule cell layer in the hippocampus. Because the rapid uptake of Aβ_1–42_ with extracellular Zn^2+^ is essential for Aβ_1–42_-induced cognitive decline *in vivo*, the uptake mechanism was tested in dentate granule cells in association with synaptic activity. *In vivo* rapid uptake of Aβ_1–42_ was not modified in the dentate granule cell layer after co-injection of Aβ_1–42_ and tetrodotoxin, a Na^+^ channel blocker, into the dentate gyrus. Both the rapid uptake of Aβ_1–42_ and Zn^2+^ into the dentate granule cell layer was not modified after co-injection of CNQX, an AMPA receptor antagonist, which blocks extracellular Zn^2+^ influx, Both the rapid uptake of Aβ_1–42_ and Zn^2+^ into the dentate granule cell layer was not also modified after either co-injection of chlorpromazine or genistein, an endocytic repressor. The present study suggests that Aβ_1–42_ and Zn^2+^ are synaptic activity-independently co-taken up into dentate granule cells in the normal brain and the co-uptake is preferential in dentate granule cells in the hippocampus. We propose a hypothesis that Zn-Aβ_1–42_ oligomers formed in the extracellular compartment are directly incorporated into neuronal plasma membranes and form Zn^2+^-permeable ion channels.

## Introduction

Alzheimer’s disease (AD) has a preclinical phase of 20–30 years prior to clinical onset and is the most common cause of dementia^1,2^. Amyloid-β (Aβ) peptides are produced by processing of amyloid precursor protein (APP)^3,4^ and have a characteristic of self-assembly into oligomers in the extracellular compartment^5^. Aβ_1–40_ and Aβ_1–42_ are the two major isoforms^6^. Aβ_1–42_ more readily forms oligomers than Aβ_1–40_ and is more neurotoxic^7^. Formation and propagation of misfolded aggregates of Aβ_1–42_ may contribute to AD pathogenesis rather than those of Aβ_1–40_. C-Terminal carboxylate anion of Aβ_1–42_, which forms the C-terminal hydrophobic core, leads to accelerate neurotoxic oligomerization^8^. C-Terminal Ala42 that is absent in Aβ_1–40_ forms a salt bridge with Lys28 and constructs a self-recognition molecular switch. The switch is the Aβ_1–42_-mediated self-replicating amyloid-propagation machinery^9^.

In AD pathogenesis, Zn^2+^ has been implicated by inducing rapid Aβ oligomerization^10–12^. The aggregation property of Aβ_1–42_ is accelerated with Zn^2+^, followed by much higher affinity of Aβ_1–42_ (K_d_ = ~3–30 nM) to Zn^2+^ than Aβ_1–40_ (K_d_ = 0.1–60 μM), which leads to neuronal Zn-Aβ_1–42_ uptake and then synaptic dysfunction. In the hippocampus, Aβ_1–42_ released into the extracellular compartment can capture Zn^2+^, which is estimated to be approximately 10 nM in the extracellular fluid^13^, even at 500 pM Aβ_1–42_ *in vivo*. We have reported that Aβ_1–42_ takes Zn^2+^ as a cargo into dentate granule cells in the normal brain, followed by cognitive decline^14–16^. Although the mechanism of Zn-Aβ_1–42_ uptake remains to be solved, extracellular Zn^2+^ is essential for Aβ_1–42_ uptake into dentate granule cells^15^.

It had been reported that Aβ oligomers form Ca^2+^-permeable plasma membrane pores that form Aβ channels, leading to a disruption of neuronal Ca^2+^ homeostasis^17,18^, which may be linked with synaptic dysfunction and neurodegeneration^19^. Aβ-mediated Ca^2+^ channels interact with Zn^2+^ and Zn^2+^ blocks extracellular Ca^2+^ influx in the range of high micromolar concentrations (>250 μM)^20^, although the gating kinetics and Ca^2+^ permeability of Aβ pores are not well understood^21^.

In the present study, we postulate that Zn-Aβ_1–42_ oligomers formed in the extracellular compartment form Zn^2+^-permeable plasma membrane pores, based on the evidence of permeation of Zn^2+^ through Ca^2+^ channels^22,23^. Here we report the mechanism on synaptic activity-independent Aβ_1–42_ uptake *in vivo*.

## Results

When Aβ_1–42_ is bound to extracellular Zn^2+^ *in vivo*, Aβ_1–42_ is rapidly taken up into dentate granule cells and affects memory via attenuated LTP^14–16^. Both uptake of Aβ_1–42_ and Zn^2+^ are observed 5 min after Aβ_1–42_ injection into the dentate gyrus, followed by Aβ toxicity and are blocked by coinjection of CaEDTA, an extracellular Zn^2+^ chelator, followed by blockade of Aβ toxicity. Thus, we need to clarify the mechanism of the rapid uptake of Aβ_1–42_ *in vivo*, which is linked with Aβ toxicity. On the other hand, endogenous Zn^2+^ released from the hippocampal slices promotes Aβ_1–42_ uptake (retention), which is determined by *in vitro* Aβ immunohistochemistry (monoclonal antibody 4G8), in the absence of additional extracellular Zn^2+^ ^15^.

**Figure 1:**
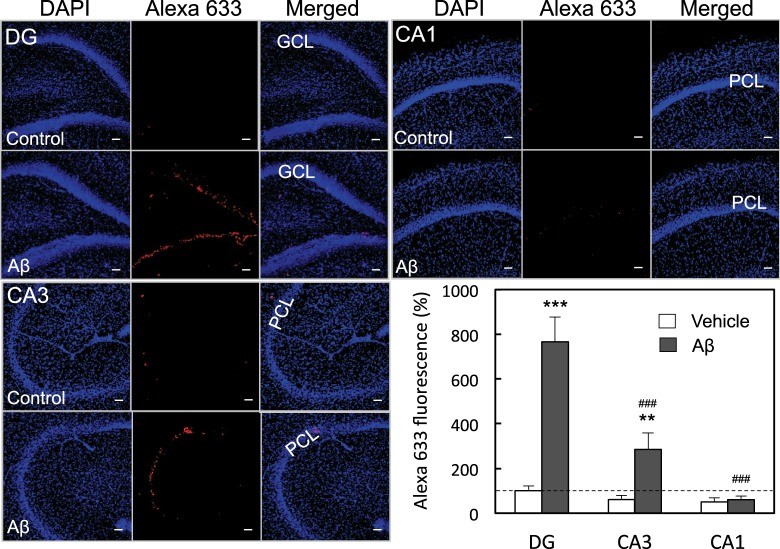
*In vitro* Aβ_1–42_ uptake in the hippocampus. Aβ immunostaining was determined in the dentate gyrus (DG), the CA3, and the CA1 15 min after incubation with 50 μM Aβ_1–42_ in ACSF. GCL, dentate granule cell layer; PCL, pyramidal cell layer. Aβ uptake in the GCL (n = 32), CA3 PCL (n = 22), and CA1 PCL (n = 22) was determined with Alexa 633 intensity, which was represented by the ratio to Alexa 633 intensity in the control GCL without 50 μM Aβ_1–42_ in ACSF expressed as 100% (lower-right). Alexa 633 intensity in the control CA3 PCL and the control CA1 PCL was also represented by the ratio to Alexa 633 intensity in the control GCL. **p < 0.01, ***p < 0.001, vs. each control with vehicle (DG, n = 29; CA3, n = 20; CA1, n = 20), ^###^p < 0.001, vs. DG with Aβ_1–42_. Bar, 50 μm.

In the present study, Aβ_1–42_ uptake was determined in rat hippocampal slices 15 min after incubation with Aβ_1–42_, Aβ_1–42_ uptake was preferentially high in the dentate granule cell layer in the hippocampus, compared with the CA3 and CA1 pyramidal cell layer (Fig. 1).

**Figure 2:**
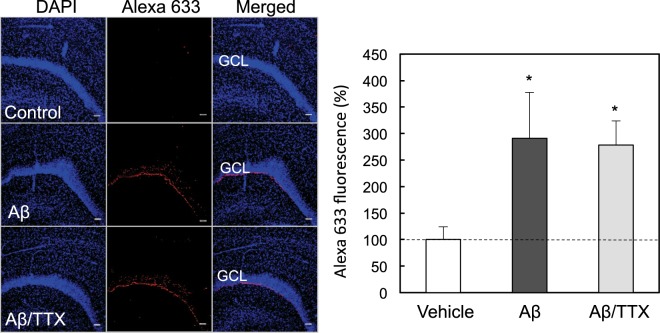
*In vivo* uptake of Aβ_1–42_ in the dentate gyrus in the presence of tetrodotoxin. Aβ_1–42_ immunostaining was determined in the dentate gyrus 5 min after injection of 50 μM Aβ_1–42_ (n = 6), and 50 μM Aβ_1–42_ + 15 μM tetrodotoxin (n = 8) in ACSF into the dentate gyrus of anesthetized rats (left). GCL, dentate granule cell layer. Bar, 50 μm. Aβ_1–42_ uptake in the dentate granule cell layer determined with Alexa 633 intensity, which is represented by the ratio to the control (n = 12) without 50 μM Aβ_1–42_ in ACSF expressed as 100%. *p < 0.05, vs. control.

To examine the involvement of neuronal activity in Aβ_1–42_ uptake, Aβ_1–42_ and tetrodotoxin, a Na^+^ channel blocker, was co-injected into the dentate gyrus of rats. The present dose (15 μM) of tetrodotoxin was determined based on the *in vivo* action as a Na^+^ channel blocker (10 μM)^24^. Aβ_1–42_ uptake was determined by *ex vivo* Aβ immunohistochemistry 5 min after the co-injection. The rapid uptake of Aβ_1–42_ was not modified in the dentate granule cell layer even by co-injection of tetrodotoxin (Fig. 2).

**Figure 3:**
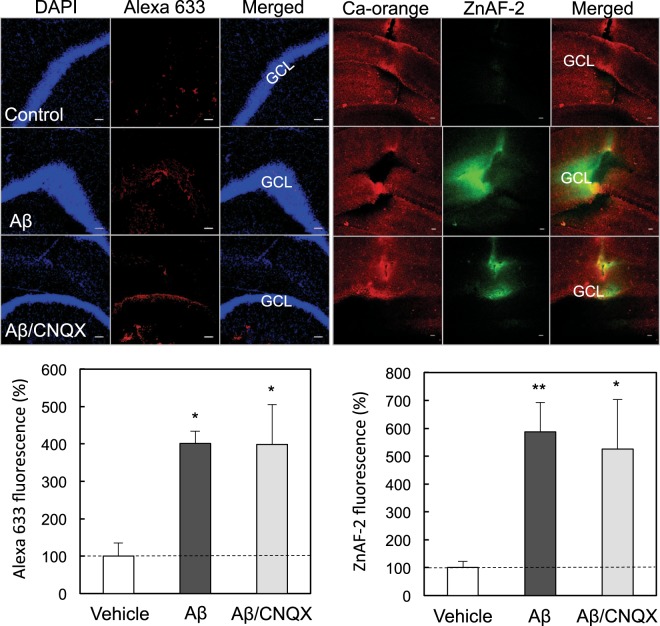
*In vivo* uptake of Aβ_1–42_ and Zn^2+^ in the dentate gyrus in the presence of CNQX. Aβ_1–42_ immunostaining (upper-left) and Zn^2+^ imaging (upper-right) were determined in the dentate gyrus 5 min after injection of 50 μM Aβ_1–42_, and 50 μM Aβ_1–42_ + 2 mM CNQX in ACSF into the dentate gyrus of anesthetized rats. GCL, dentate granule cell layer. Bar, 50 μm. Aβ_1–42_ uptake in the dentate granule cell layer determined with Alexa 633 intensity, which is represented by the ratio to the control (control, n = 6; Aβ, n = 5; Aβ/CNQX, n = 6) without 50 μM Aβ_1–42_ in ACSF expressed as 100% (lower-left). Zn^2+^ uptake in the dentate granule cell layer determined with ZnAF-2 intensity, which is represented by the ratio to the control (control, n = 11; Aβ, n = 10; Aβ/CNQX, n = 6) without 50 μM Aβ_1–42_ in ACSF expressed as 100% (lower-right). *p < 0.05, **p < 0.01, vs. control.

AMPA receptor activation induces extracellular Zn^2+^ influx *in vivo*, which is blocked in the presence of 2 mM CNQX, and the excess influx leads to cognitive decline via attenuated LTP^25,26^. The present dose of CNQX (2 mM) was used according to the previous papers. Both the rapid uptake of Aβ_1–42_ and Zn^2+^ into the dentate granule cell layer was not modified by co-injection of CNQX (Fig. 3), suggesting Aβ_1–42_-mediated extracellular Zn^2+^ influx.

**Figure 4:**
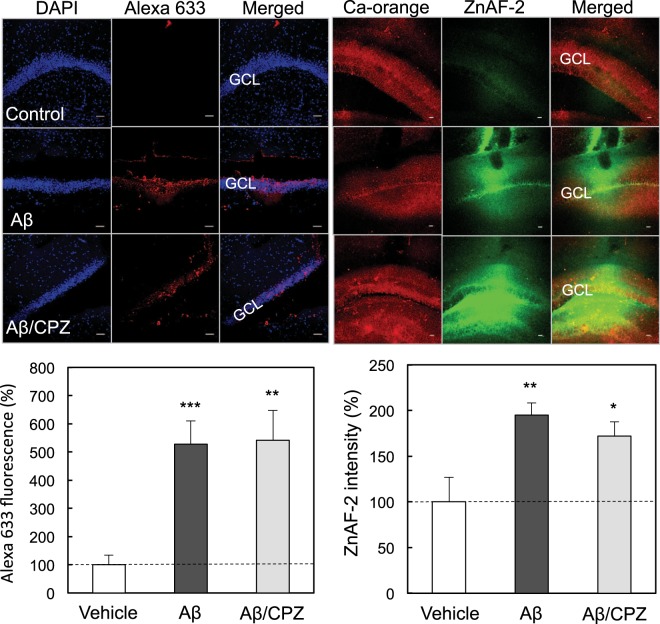
*In vivo* uptake of Aβ_1–42_ and Zn^2+^ in the dentate gyrus in the presence of chlorpromazine (CPZ). Aβ_1–42_ immunostaining (upper-left) and Zn^2+^ imaging (upper-right) were determined in the dentate gyrus 5 min after injection of 50 μM Aβ_1–42_, and 50 μM Aβ_1–42_ + 500 μM chlorpromazine in ACSF into the dentate gyrus of anesthetized rats. GCL, dentate granule cell layer. Bar, 50 μm. Aβ_1–42_ uptake in the dentate granule cell layer determined with Alexa 633 intensity, which is represented by the ratio to the control (control, n = 11; Aβ, n = 10; Aβ/CPZ, n = 5) without 50 μM Aβ_1–42_ in ACSF expressed as 100% (lower-left). Zn^2+^ uptake in the dentate granule cell layer determined with ZnAF-2 intensity, which is represented by the ratio to the control (control, n = 10; Aβ, n = 9; Aβ/CNQX, n = 11) without 50 μM Aβ_1–42_ in ACSF expressed as 100% (lower-right). *p < 0.05, **p < 0.01, ***p < 0.001, vs. control.

**Figure 5:**
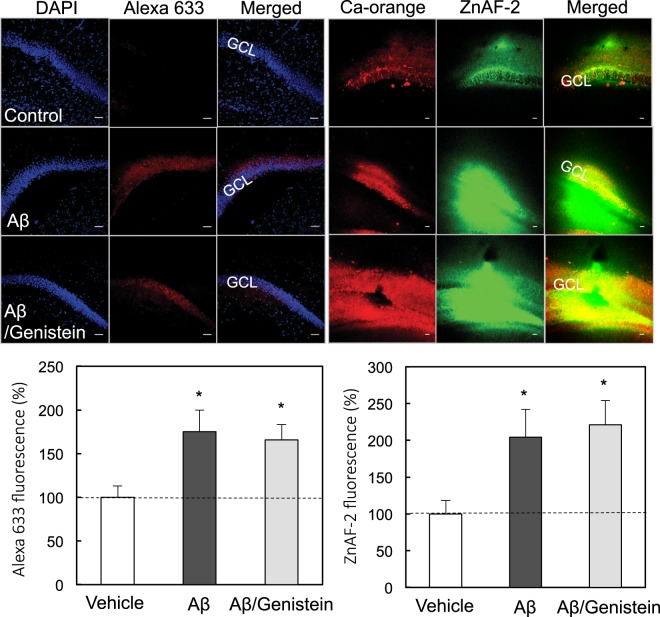
*In vivo* uptake of Aβ_1–42_ and Zn^2+^ in the dentate gyrus in the presence of genistein. Aβ_1–42_ immunostaining (upper-left) and Zn^2+^ imaging (upper-right) were determined in the dentate gyrus 5 min after injection of 50 μM Aβ_1–42_, and 50 μM Aβ_1–42_ + 250 μM genistein in ACSF into the dentate gyrus of anesthetized rats. GCL, dentate granule cell layer. Bar, 50 μm. Aβ_1–42_ uptake in the dentate granule cell layer determined with Alexa 633 intensity, which is represented by the ratio to the control (control, n = 5; Aβ, n = 4; Aβ/genistein, n = 6) without 50 μM Aβ_1–42_ in ACSF expressed as 100% (lower-left). Zn^2+^ uptake in the dentate granule cell layer determined with ZnAF-2 intensity, which is represented by the ratio to the control (control, n = 8; Aβ, n = 5; Aβ/genistein, n = 5) without 50 μM Aβ_1–42_ in ACSF expressed as 100% (lower-right). *p < 0.05, vs. control.

We examined whether endocytosis is involved in the rapid uptake of Aβ_1–42_ and Zn^2+^. Chlorpromazine (500 μM), a clathrin/caveolae-mediated endocytosis blocker, was used based on the *in vivo* action as a blocker (200 μM)^27^. Genistein (250 μM), a caveolae/raft-mediated endocytosis blocker, was used based on the blocking effect on the primary entry pathway for aquareovirus (<200 μM)^28^. Both the rapid uptake of Aβ_1–42_ and Zn^2+^ into the dentate granule cell layer was not also modified by either co-injection of chlorpromazine (Fig. 4) or genistein (Fig. 5).

## Discussion

*In vivo* LTP at medial perforant pathway-dentate granule cell synapses, which is closely linked to object recognition memory^29^, is not affected even under perfusion with 1,000 nM Aβ_1–42_ in ACSF without Zn^2+^, but attenuated under pre-perfusion with 500 pM Aβ_1–42_ in ACSF containing 10 nM Zn^2+^. The attenuation is rescued by extracellular Zn^2+^-chelation with CaEDTA. These data indicate that high picomolar Aβ_1–42_ captures extracellular Zn^2+^ and subsequently attenuates LTP^15^. The evidence is consistent with Aβ_1–42_-induced object recognition memory deficit, which is also rescued by CaEDTA and ZnAF-2DA, an intracellular Zn^2+^ chelator^13^. Thus, extracellular Zn^2+^ is essential for Aβ_1–42_-induced cognitive decline via attenuated LTP in the normal brain, consistent with *in vivo* complete blocking rapid uptake of Aβ_1–42_ and Zn^2+^ into dentate granule cells after co-injection of Aβ_1–42_ and CaEDTA into the dentate gyrus^15^. In the present study, we tested the uptake mechanism of Aβ_1–42_ oligomers *in vitro* and *in vivo*.

When Aβ_1–42_ uptake was determined in hippocampal slices after incubation with Aβ_1–42_, Aβ_1–42_ uptake was preferentially high in the dentate granule cell layer in the hippocampus. Aβ_1–42_ uptake was also considerably high in the CA3 pyramidal cell layer, while Aβ_1–42_ uptake was not observed in the CA1 pyramidal cell layer. Extracellular Zn^2+^ interaction with Aβ_1–42_ is essential for Aβ_1–42_ uptake into dentate granule cells *in vivo* and its essentiality is the same even under *in vitro* hippocampal slice condition^15^. Endogenous Zn^2+^ release from the hippocampal slices is required for the Aβ_1–42_ uptake. In hippocampal slices bathed in ACSF without Zn^2+^, extracellular Zn^2+^ level determined with ZnAF-2 is the highest in the hilus close to the dentate granule cell layer and the second highest in the stratum lucidum close to the CA3 pyramidal cell layer^30^. Endogenous Zn^2+^, which is released from mossy fibers, may be closely linked with the Aβ_1–42_ uptake. Although the Schaffer collaterals also release Zn^2+^, it is estimated that its release is not enough for the Aβ_1–42_ uptake into CA1 pyramidal cells *in vitro*. In contrast, it is estimated that Aβ_1–42_ captures extracellular Zn^2+^ and is taken up into CA1 pyramidal cell *in vivo*. *In vivo* CA1 LTP is impaired after intracerebroventricular injections of Aβ peptide fragments^31^, implying that extracellular Zn^2+^ is potentially involved in the impairment. Intracellular infusion of oligomerised Aβ_1–42_ via passive diffusion from the patch pipette induces the rapid synaptic insertion of Ca^2+^-permeable AMPA receptors in CA1 pyramidal cells^32^. When oligomeric Aβ_1–42_ captures extracellular Zn^2+^ *in vivo*, it is also possible that intracellular oligomeric Aβ_1–42_ affect neuronal functions. Postmortem studies suggest that the hippocampus and entorhinal cortex are the first brain regions to be affected in Alzheimer’s disease^1,33,34^. The perforant pathway-dentate granule cell synapses are vulnerable to Aβ synapse toxicity^35^, which may be linked with extracellular Zn^2+^-mediated Aβ_1–42_ uptake into dentate granule cells. This uptake may be associated with the high level of extracellular Zn^2+^ in the hilus that may be due to Zn^2+^ release form mossy fibers.

The mechanism of Aβ_1–42_ uptake was tested in the dentate granule cell layer *in vivo*. When Aβ_1–42_ was co-injected with tetrodotoxin, a Na^+^ channel blocker, into the dentate gyrus, the rapid uptake of Aβ_1–42_ was not modified in the dentate granule cell layer. AMPA receptor activation induces extracellular Zn^2+^ influx and the excess influx leads to cognitive decline via attenuated LTP^25,26^. In contrast, both the rapid uptake of Aβ_1–42_ and Zn^2+^ into the dentate granule cell layer was not modified after co-injection of CNQX, an AMPA receptor antagonist, suggesting that Zn-Aβ_1–42_ oligomers are ionotropic glutamate receptor activation-independently taken up into dentate granule cells. Both the rapid uptake of Aβ_1–42_ and Zn^2+^ into the dentate granule cell layer was not also modified after either co-injection of chlorpromazine or genistein, an endocytic repressor. It is likely that Zn-Aβ_1–42_ oligomers are rapidly taken up into dentate granule cells without interaction with plasma membrane receptor proteins.

In human SH-SY5Y neuroblastoma, monomeric Aβ_1–42_ is selectively internalized via clathrin- and dynamin-independent endocytosis compared to monomeric Aβ_1–40_ ^36^. Genistein, a major phytoestrogen in soybean, may reduce the Aβ_1–42_-induced cell toxicity by suppressing the formation of toxic, cell membrane penetrating Aβ_1–42_ oligomers in human SH-SY5Y neuroblastoma^37^. Zn^2+^ interaction with Aβ_1–42_ has not been taken into account in these *in vitro* cell culture systems, suggesting that Zn-Aβ_1–42_ oligomers formed *in vivo* are taken up into dentate granule cells via a novel mechanism and causes cytotoxicity. On the other hand, Aβ production occurs in acidic vesicular organelles and contributes both to Aβ secretion and to the direct accumulation of Aβ within neurons^37^. Intracellular Aβ produced by the direct accumulation can exist as a monomeric form that further aggregates into oligomers and it may mediate pathological events^38^, although it is unknown whether Zn^2+^ is involved in the pathological effects.

The Aβ ion channel hypothesis has been reported in AD pathogenesis. These authors have reported that Aβ peptides disrupt Ca^2+^ homeostasis in neurons and increase intracellular Ca^2+^ level, resulting in synaptic dysfunction and neurodegeneration. The hypothesis is based on the results from *in vitro* experimental systems such as artificial membranes and neuronal culture^18,39^. Judging from capturing extracellular Zn^2+^ with high picomolar (100–500 pM) Aβ_1–42_ in the hippocampal extracellular compartment, which disrupts Zn^2+^ homeostasis in neurons and increases intracellular Zn^2+^ level^14^, the Aβ-mediated Ca^2+^ channel hypothesis might be changed into the Aβ_1–42_-mediated Zn^2+^ channel hypothesis in AD pathogenesis. Because Aβ stain is observed around the nuclei in dentate granule cells^15^, a portion of Aβ_1–42_ is co-taken up with Zn^2+^ into dentate granule cells even if Aβ_1–42_-mediated Zn^2+^ channels (pores) is formed in the plasma membrane.

In conclusion, the present study suggests that amyloid β_1–42_ and Zn^2+^ are synaptic activity-independently co-taken up into dentate granule cells in the normal brain and the co-uptake is preferential in dentate granule cells. We propose a hypothesis that Zn-Aβ oligomers formed in the extracellular compartment are directly incorporated into neuronal membranes and form Zn^2+^-permeable ion channels. Because extracellular Zn^2+^ is age-relatedly increased in the rat hippocampus^40^, Zn-Aβ_1–42_ oligomers are more readily produced in the extracellular compartment of the aged hippocampus, followed by vulnerability to Zn-Aβ_1–42_ oligomers in aging^16,41^. Therefore, controlling intracellular Zn^2+^ dysregulation may be an effective strategy for overcoming AD pathogenesis.

## Experimental Procedures

### Animals and chemicals

Wistar rats (male, 7–9 weeks of age) were obtained from Japan SLC (Hamamatsu, Japan). Rats were housed under the standard laboratory conditions (23 ± 1 °C, 55 ± 5% humidity) and had access to water and food *ad libitum*. Rats were used for experiments approximately 1 week after housing. All the experiments were performed according to the Guidelines for the Care and Use of Laboratory Animals in the University of Shizuoka, which refer to the American Association for Laboratory Animals Science and the guidelines laid down by the NIH (NIH Guide for the Care and Use of Laboratory Animals) in the USA. The present study has been approved by the Ethics Committee for Experimental Animals in the University of Shizuoka.

Synthetic human Aβ_1–42_ was purchased from ChinaPeptides (Shanghai, China). Aβ was dissolved in saline at the time of need. Aβ_1–42_ prepared in saline was mainly monomers with a small fraction of low order oligomers in SDS-PAGE^14^. ZnAF-2DA (K_d_ = 2.7 × 10^−9^ M for Zn^2+^) is a membrane-permeable Zn^2+^ indicator and was kindly supplied from Sekisui Medical Co., LTD (Hachimantai, Japan). This indicator is taken up into the cells through the plasma membrane and is hydrolyzed by esterase in the cytosol to produce ZnAF-2, which does not permeate the plasma membrane^42,43^. ZnAF-2 is selectively bound to Zn^2+^, but not bound to other divalent cations such as Ca^2+^, Mg^2+^, and Cu^2+^ ^42^. Calcium Orange AM is a membrane-permeable Ca^2+^ indicator and was purchased from Molecular Probes, Inc. (Eugene, OR). The fluorescence indicators were dissolved in dimethyl sulfoxide and diluted to artificial cerebrospinal fluid (ACSF) containing 119 mM NaCl, 2.5 mM KCl, 1.3 mM MgSO_4_, 1.0 mM NaH_2_PO_4_, 2.5 mM CaCl_2_, 26.2 mM NaHCO_3_, and 11 mM D-glucose (pH 7.3).

### Hippocampal slice preparation

Rats were anesthetized with ether and decapitated under anesthesia. The brain was quickly removed from rats and bathed in ice-cold choline-ACSF, which consists of 124 mM choline chloride, 2.5 mM KCl, 2.5 mM MgCl_2_, 1.25 mM NaH_2_PO_4_, 0.5 mM CaCl_2_, 26 mM NaHCO_3_, and 10 mM glucose (pH 7.3) to block excessive neuronal excitation. Horizontal hippocampal slices (400 μm) were prepared in ice-cold choline-ACSF by using a vibratome ZERO-1 (Dosaka Kyoto, Japan). Hippocampal slices were placed in ACSF at 25 °C for at least 30 minutes. All solutions were continuously bubbled with 95% O_2_ and 5% CO_2_.

### *In vitro* immunostaining

Hippocampal slices were bathed in 50 μM Aβ_1–42_ in ACSF for 15 min. Slices were then rinsed twice with ACSF for 5 min to remove extracellular agents, and fixed with paraformaldehyde (4% in 0.01 M PBS) for 15 min. Slices were rinsed in 0.01 M PBS three times. Slice tissues were blocked in 10% normal goat serum for 30 min, followed by rinse in 0.01 M PBS three times, incubated with 70% formic acid for 5 min, rinsed with 0.01 M PBS three times, and bathed at 4 °C in Aβ monoclonal antibody, 4G8 (COVANCE, 1:500 dilution in 0.01 M PBS) for 48 h. Slices were then rinsed with 0.01 M PBS three times, bathed in Alexa Fluor 633 goat anti-mouse IgG secondary antibody (1: 200 dilution in 0.01 M PBS) for 1 h, rinsed with 0.01 M PBS three times, bathed in 4′,6-diamidino-2-phenylindole (DAPI) for 10 min, rinsed again with 0.01 M PBS three times, and mounted on glass slides. Immunostaining images were obtained by using a confocal laser-scanning microscopic system (Nikon A1 confocal microscopes, Nikon Corp.) through a 10× objective. Florescence intensity was analyzed by the NIH Image J.

### *Ex vivo* immunostaining

Rats anesthetized with chloral hydrate (400 mg/kg) were placed in a stereotaxic apparatus. The skull was exposed and two burr holes were drilled. Injection cannulae (internal diameter, 0.15 mm; outer diameter, 0.35 mm) were bilaterally inserted into the dentate granule cell layer (4.0 mm posterior to the bregma, 2.3 mm lateral, 2.9 mm inferior to the dura) of the anesthetized rats. Thirty minutes later following recovery from the insertion damage, 50 μM Aβ_1–42_ in ACSF, or 50 μM Aβ_1–42_ with either 15 μM tetrodotoxin, a Na^2+^ channel blocker, 2 mM 6-cyano-7-nitroquinoxaline-2,3-dione (CNQX), an α-amino-3-hydroxy-5-methyl-4-isoxazolepropionate (AMPA) receptor antagonist, 500 μM chlorpromazine, a clathrin/caveolae-mediated endocytosis blocker, or 250 μM genistein, a caveolae/raft-mediated endocytosis blocker, in ACSF were bilaterally injected into the dentate granule cell layer at the rate of 0.25 μl/min for 8 min via the injection cannulae. Five minutes later, the brain was quickly removed from the rats and immunostaining images in hippocampal slices were obtained in the same manner except for changing the 10% goat serum with 5% goat serum. The positions of the injection cannulae were confirmed in the slice preparation.

### *In vivo* intracellular Zn^2+^ imaging

Fifty μM Aβ_1–42_ in ACSF containing 100 μM ZnAF-2DA, or 50 μM Aβ_1–42_ with either 15 μM tetrodotoxin, 2 mM CNQX, 500 μM chlorpromazine, or 250 μM genistein in ACSF containing 100 μM ZnAF-2DA was bilaterally injected via injection cannulae into the dentate granule cell layer of anesthetized rats at the rate of 0.25 μl/min for 8 min. Five minutes later, the hippocampal slices were prepared in the same manner. Slices were transferred to a chamber filled with ACSF, loaded with 2 μM Calcium Orange AM in ACSF for 30 min, and then rinsed in ACSF for 30 min. The hippocampal slices were transferred to a recording chamber filled with ACSF. The fluorescence of ZnAF-2 (laser, 488.4 nm; emission, 500–550 nm), and calcium orange (laser, 561.4 nm; emission, 570–620 nm) was measured with a confocal laser-scanning microscopic system (Nikon A1 confocal microscopes, Nikon Corp.). Calcium Orange AM was used to identify hippocampal regions in slices. The positions of the injection cannulae were confirmed in the slice preparation.

### Data analysis

For multiple comparisons, differences between treated groups were analyzed by one-way ANOVA followed by post hoc testing using the Tukey’s test. A value of p < 0.05 was considered significant (the statistical software, GraphPad Prism 5). Data were expressed as means ± standard error. The control group with vehicle was compared with the treated groups in all figures for the statistical analysis. In Fig. 1, Aβ group in the DG was also compared with those in the CA3 and the CA1.

### Ethics statement

All experiments were done according to the Guidelines for the Care and Use of Laboratory Animals of the University of Shizuoka, which refer to American Association for Laboratory Animals Science and the guidelines laid down by the NIH (NIH Guide for the Care and Use of Laboratory Animals) in the USA. All experimental protocols were approved by the ethics committee of the University of Shizuoka
